# Supplementary figures and images for: Dexamethasone-Induced Fatty Acid Oxidation and Autophagy/Mitophagy Are Essential for T-ALL Glucocorticoid Resistance
Source: Cancers (Basel). 2023 Jan 10;15(2):445. doi: 10.3390/cancers15020445 (PMC9856638; doi:10.3390/cancers15020445)

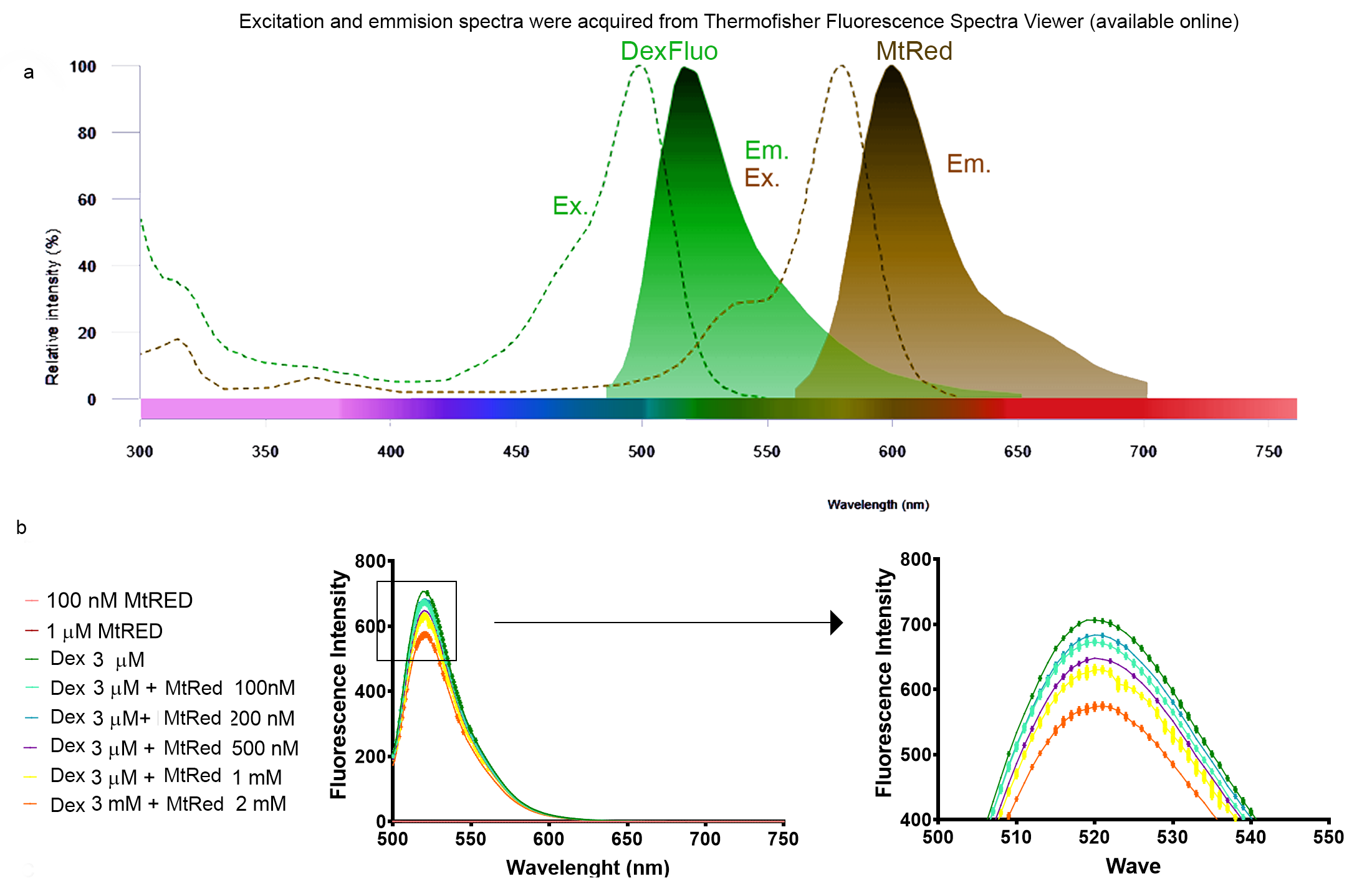

Supplement: Supplementary file 1 [file cancers-15-00445-s001.zip › Figure S1.tif]

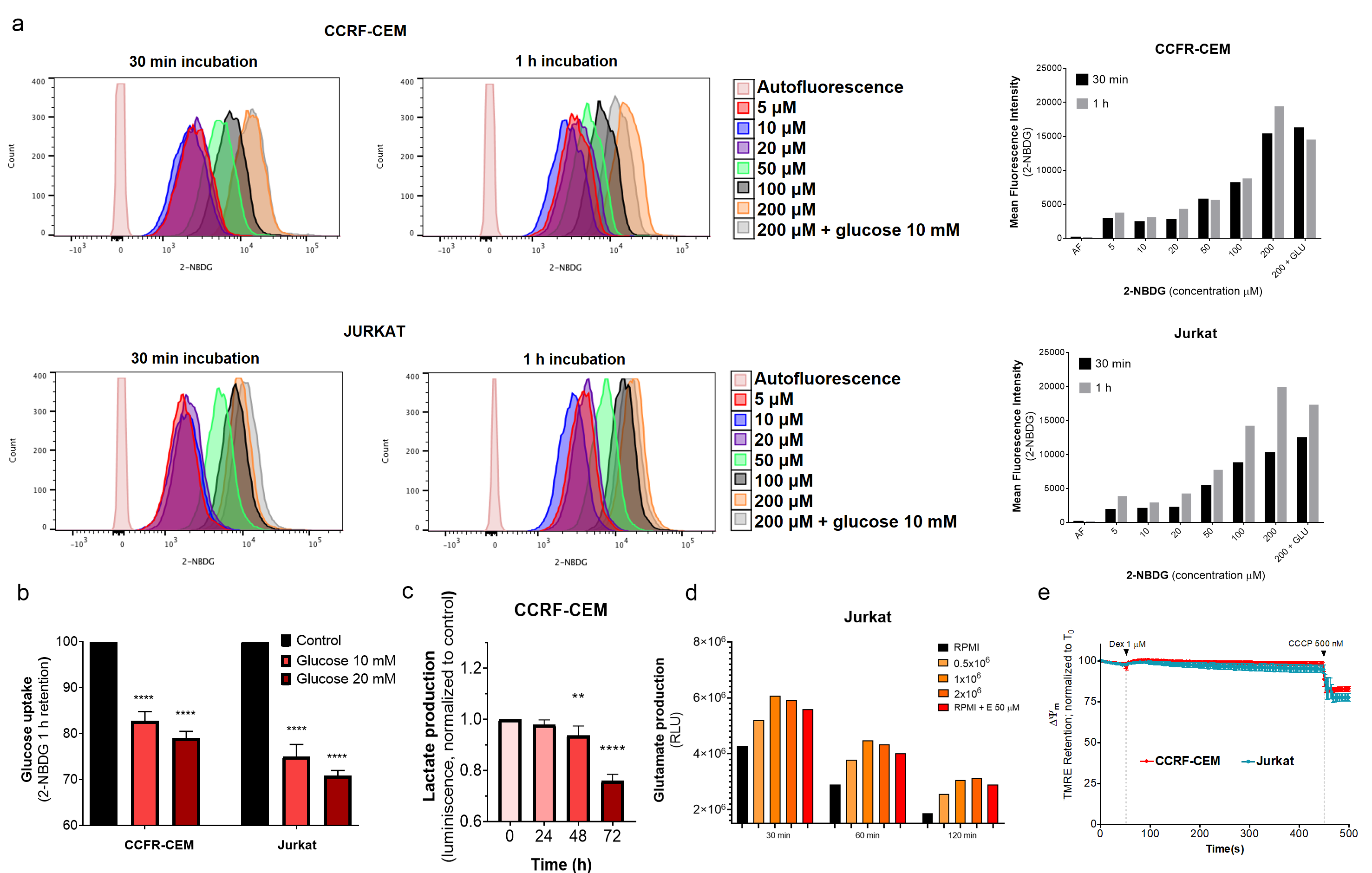

Supplement: Supplementary file 1 [file cancers-15-00445-s001.zip › Figure S2.tif]
